# Rel/Nuclear factor-kappa B apoptosis pathways in human cervical cancer cells

**DOI:** 10.1186/1475-2867-5-10

**Published:** 2005-04-27

**Authors:** Marlene F Shehata

**Affiliations:** 1 Division of Basic Sciences, Faculty of Medicine, Memorial University of Newfoundland, St. John's, A1B 3V6, Canada

**Keywords:** Cervical cancer, HeLa cells, NF-κB, Xrel3, cisplatin, apoptosis, anti-apoptosis

## Abstract

Cervical cancer is considered a common yet preventable cause of death in women. It has been estimated that about 420 women out of the 1400 women diagnosed with cervical cancer will die during 5 years from diagnosis. This review addresses the pathogenesis of cervical cancer in humans with a special emphasis on the human papilloma virus as a predominant cause of cervical cancer in humans. The current understanding of apoptosis and regulators of apoptosis as well as their implication in carcinogenesis will follow. A special focus will be given to the role of Rel/NF-κB family of genes in the growth and chemotherapeutic treatment of the malignant HeLa cervical cells emphasizing on Xrel3, a cRel homologue.

## Introduction

### A. Oncogenesis

The process of oncogenesis or carcinogenesis fundamentally emerges from defects in the balance between the activity of proto-oncogenes, which promote cell proliferation, and tumor suppressor genes, which regulate the cell cycle. It is known that DNA damage and repair occurs normally in every living cell. When the rate of DNA damage exceeds that of repair, accumulation of DNA damage and defects might trigger the initiation of cancer [[1-3] and [4]].

Uterine cervical cancer is a serious gynecologic malignancy in women. There are two main types of cervical cancer, squamous cell cancer and adenocarcinoma, based on the type of cells that become cancerous. Cervical cancer is initiated when the combined action of a group of carcinogens cause the normal, physiological events associated with cervical metaplastic transformation to go awry and cause the formation of pre-malignant dysplasia [5]. Poor prognosis is usually associated with positive pelvic lymph nodes, indicating that the tumor cells have become metastatic [6].

Recent studies have demonstrated that estrogen, which is the female sex hormone, might have a contributory role in increasing vaginal epithelium proliferation and thus promoting the malignant transformation of the squamous and columnar cells at the junction of the cervical and vaginal epithelium [7]. Infection by the Human Papilloma Virus, HPV, is a necessary requirement for cervical cancer, but not all women infected by this virus develop cervical cancer [8]. Some HPV infections, for instance are associated with benign proliferation or wart formation.

### B. Human Papilloma Virus (HPV)

HPVs are small DNA viruses that are known to be the most common etiological agents in cervical cancer [9]. More than 100 types of HPVs have been discovered, isolated and studied (See Table 1) [10]. HPVs are implicated in the mucosal and epithelial infections that may range from a benign lesion to a malignant carcinoma [4]. HPV has also been reported to be associated with anal and genital cancers [11]. Preliminary findings suggested their involvement in some head and neck cancers as well [10].

**Table 1:** Naturally occurring cancers associated with papillomaviruses [10, 13].

**Species**	**Cancer**	**Predominant viral types**
Humans	Skin carcinomas	HPV-5, -8
	Lower genital tract cancers	HPV-16, -18, -31, -33
	Malignant progression of respiratory papillomas	HPV-6, -11
Cattle	Alimentary-tract carcinoma	HPV-4
	Eye and skin carcinoma	Not characterized
Sheep	Skin carcinoma	Not characterized
Cottontail rabbit	Skin carcinoma	Cotton rabbit papillomavirus (CRPV)

**Figure 1:**
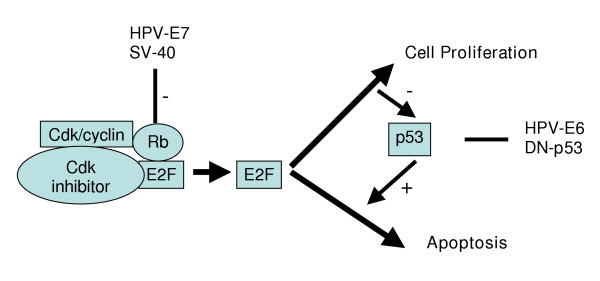
**A schematic representation of RB/p53 interactions to regulate cell cycle and apoptosis**. Cell cycle transition from G_1_-S phase is mediated by RB interactions with the E2F transcription factor family, which is considered an important regulator of the cell cycle. Growth factors lead to the phosphorylation of RB in late G_1 _phase by cdk/cyclin. This is followed by the release of E2F, allowing transcriptional activation of E2F target genes, which promotes S-phase entry and cell proliferation. HPV E7 and Simian Virus 40 (SV40) promote the release of E2F from RB, whereas HPV E6 and the dominant negative, DN-p53 inhibit p53 activity leading to cell proliferation.

The high risk HPV 16 and HPV 18 are associated with malignant transformation and carcinogenesis in 85% of the diagnosed cervical cancer cases [4]. Recent studies have shown that 13 different types of HPV are associated with carcinogenesis [3]. The most widely known factors associated with HPV are the E6 and E7 oncoproteins, which interact with p53 and Rb tumor suppressors respectively [2]. The interaction of E6 and E7 with these cellular proteins results in their suppression [9], thus disrupting the normal physiological process of programmed cell death in response to DNA damage (See Figure 1) [12]. In the presence of carcinogens, therefore, the accumulation of DNA damage without apoptosis is presumed to lead to cancer.

It should be made clear that viral infection by itself does not cause cancer. It is the interaction of the viral genome with host genes that disrupts the normal cell cycle and transforms the cell into a pre-malignant state. For instance, some viruses might interact with specific genes (like tumor suppressor genes mentioned above) in the host cells, switching some systems on or off, thus leaving the cell free to divide in an uncontrolled way and raising the risk of cancer [13].

Other cellular proteins may be affected by HPV infection as well. For instance, cervical cancer cell lines showed overexpression of the anti-apoptotic protein BAG-1, which might contribute to its malignant proliferation [14]. Therefore, understanding the molecular mechanisms leading to this disease will be of importance for generating means for its early detection and possible prevention and treatment.

### C. Apoptosis

Apoptosis, or programmed cell death, is orchestrated by a highly organized group of signaling pathway proteins [[15] and [16]]. Apoptosis can be triggered by a variety of events the cell may face. For example, exposures to X-rays, ultraviolet light and chemotherapeutic drugs are factors that can initiate the process of apoptosis [15].

One mechanism to protect an organism from the consequences of accumulated DNA damage involves a class of protein-splitting enzymes called caspases, which are activated upon detection of DNA damage and eventually cause cell death [17]. The control of programmed or physiological cell death acts as a protective mechanism for the organism because accumulation of DNA damage without concomitant repair could lead to the development of cancer, while unregulated apoptosis can cause autoimmune diseases.

The process of apoptosis is essential in stopping the uncontrolled proliferation of cells [18]. Any defects in this dynamic process may eventually lead to the development of benign proliferative lesions or even malignant tumors [18]. Apoptosis can be initiated via specific receptors that are members of the tumor necrosis factor (TNF) receptor superfamily [19]. Such "Death receptors" are activated via binding of specific ligands [19] and once they are activated, they can initiate apoptosis. However, the role of these receptors is not restricted to the initiation of apoptosis, but also includes other functions that differ from apoptosis and sometimes counteract apoptosis such as the recruitment and subsequent ligand binding of growth factors such as the Nerve Growth Factor (NGF) [19].

Another apoptotic pathway involves the mitochondria. Under stress, these essential organelles can release cytochrome c into the cytoplasm of the cell [20]. This cytochrome c release is a possible activator for caspases by the recruitment of procaspase-9, which undergoes conformational changes that leads to the activation of downstream, effector caspases [20].

Apoptosis acts as a double-edged sword. Despite its importance in restricting cell proliferation and maintaining constant cell number, excessive apoptosis is associated with stroke, Alzheimer's disease and other neurodegenerative disorders [21]. Damaged neurons in these disorders commit suicide inappropriately. Alzheimer's disease, for instance, was found to be associated with a genetic component that involves mutations in the chromosomes (1, 14, and 21) as well as the tau gene on chromosome 17 and results in unscheduled or unregulated death of brain cells [22].

Understanding the details and the signaling pathways of this phenomenon might be helpful in manipulating and intervening in the process of apoptosis. Apoptosis is required to restrict cell proliferation and to maintain a constant cell number. Attempts to suppress apoptosis, however, may be useful to treat neurodegenerative disorders, while attempts to activate apoptosis may be useful in disorders involving overproliferation.

### D. Regulators of apoptosis

Many genes have been implicated in enhancing or inhibiting the process of apoptosis. They act by different mechanisms that ultimately contribute to either tumor suppression or progression, respectively. Four of the most important factors that regulate apoptosis are p53, the caspases, the Bcl-2 family of proteins and PARP [[23,24], and [25]].

#### 1. Caspases

Caspases are the executioners of cell death. They receive the signals that enable them to initiate apoptosis. Cells undergoing apoptosis exhibit fragmentation of DNA, condensation of the chromatin, budding of the cell membrane and the formation of apoptotic bodies by dissociation of the cell and its constituents into membrane-enclosed vesicles [[17] and [26]]. All caspases share a similar structure that consists of three domains: an NH_2_-terminal peptide (prodomain), a large subunit (approximately 20 kD) and a small subunit (approximately 10 kD) [27]. Caspases are expressed as procaspases, which undergo cleavage to the 2 subunits mentioned above [28]. Cleavage of caspases is a sign of active apoptosis. The large and small subunits then associate to form a heterodimer [[29] and [23]].

The exact mechanism of action of caspases is still unknown. However, several studies have shown that caspases exert both direct and indirect actions on the cell [30]. The direct action of caspases can be exemplified by their ability to act on cell structural integrity by destroying the nuclear lamina [30] and cleaving the proteins responsible for regulating the cytoskeleton [[30] and [31]]. The indirect action of caspases is via their ability to inhibit the proteins that promote cell survival and growth [27]. Among these proteins is the Bcl-2 family of proteins, which are cleaved by caspases resulting in inactivation of the Bcl-2 proteins and the release of a fragment that has a direct apoptotic effect [30].

Caspases-8, -9, and -10 are known to initiate the caspase activation cascade. However, caspases-3, -6 and -7 propagate the cascade and are activated by the proteolytic cleavage process mediated by other upstream caspases in the caspase cascade pathway [27].

#### 2. Bcl-2 family of proteins

The Bcl-2 family of proteins has several members with various functions [18]. The Bcl-2 gene family comprises pro-apoptotic and anti-apoptotic proteins sharing one or more Bcl-2 homology (BH) domains [23]. The gene family is made up of 3 main groups [27]. Group I includes the anti-apoptotic members similar to Bcl-2. Group II includes the pro-apoptotic members like Bax and Bak, while group III comprises a diverse collection of proteins that resemble one another structurally, but not necessarily functionally. Bcl-2, BAG-1 and Bcl-x_L _provide a cell survival function [[14] and [32]]. However, Bax, which promotes apoptosis translocates to the mitochondrial membrane and releases cytochrome c, which can initiate the apoptotic cascade [33]. It also competes for binding with Bcl-2 and with other members of the Bcl-2 superfamily of proteins [33]. Such heterodimerization between anti-apoptotic and pro-apoptotic members of this family is very common and is considered a regulatory mechanism for the decision to undergo apoptosis [23]. Thus, the balance between Bcl-2 and Bax is essential for the determination of the apoptotic potential of the cells, in which high apoptotic activity is often associated with a low Bcl-2/Bax level ratio [23].

BAG-1 has been shown to provide an anti-apoptotic effect. Its overexpression in cervical cancer suppressed apoptosis both independently and by increasing Bcl-2 protective activity, which further increased the resistance of cervical carcinoma to the effect of DNA-damaging agents [14].

#### 3. p53

The tumor suppressor protein p53 has numerous functions (See Figure 1.2) [34]. Its principle role, however, is as a transcriptional regulator required for the expression of a number of genes involved in cell cycle regulation and apoptosis. The gene encoding p53 can be mutated in many forms of cancer including cervical, uterine, adenocarcinoma, adrenal and colorectal cancers. In cervical cancer, mutation patterns of p53 may vary from point mutation to deletion to base-pair alteration, however 30% of the cases showed a higher percentage of Guanine-Cytosine complementary base pairs compared to the Adenine-Thymine complementary base pairs suggesting that alteration in the base-pairing sequence is the major mutation pattern recognized in p53 [34]. A recent clinical study showed that the overexpression of p53 in cisplatin-treated tumors might be associated with resistance of the tumor to further cell death and apoptosis [35,36].

MDM2 is a p53-regulated protein that has a role in the translocation of p53 from the nucleus and enhances its proteosomal degradation [22]. Therefore, increased levels of MDM-2 and subsequent low levels of p53 are associated with increased cell growth and proliferation. The p53 tumor suppressor protein can also be targeted for degradation by the E6 oncogene of the Human Papilloma virus (HPV), thus promoting neoplastic proliferations (refer to Figure 1) [34].

#### 4. PARP

**Figure 2:**
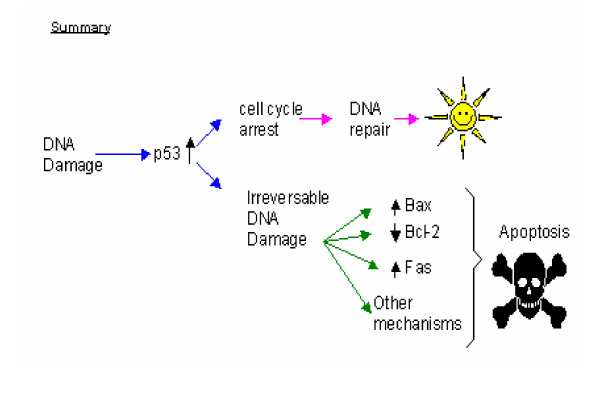
**Summary of the mechanism of action of the tumor suppressor protein, p53**. After DNA damage, the tumor suppressor protein, p53, will be upregulated causing cell cycle arrest and enhancing DNA repair. However, in cases of irreversible DNA damage, p53 has been shown to transcriptionally repress the antiapoptotic gene Bcl-2, while it upregulates the pro-apoptotic proteins Bax and Fas. This in turn, promotes apoptosis. During apoptosis loss of the integrity of the mitochondrial membrane is followed by release of cytochrome c into the cytosol, this in turn leads to activation of caspase cleavage. Bax has been shown to contain p53-binding sites in its promoter site and is upregulated in response to DNA damage and increased p53 [36].

Poly (ADP-ribose) polymerase, PARP, has recently been found to promote cell death, but the exact mechanism of action of PARP remains largely obscure. Many cellular enzymes were found to contain the PARP catalytic subunit, but they have different cellular localizations. Because PARP activation consumes much cellular energy, detection of abnormally high levels of PARP in cells might indicate excessive energy consumption and cellular exhaustion [22]. PARP is also known as an apoptosis-inducing factor and high levels of PARP are detected following DNA damage. Thus, this group of enzymes might also be involved in DNA repair, as well as apoptotic responses of the cells (summarized in Figure 2) [22].

### E. Rel/NF-κB family

**Figure 3:**
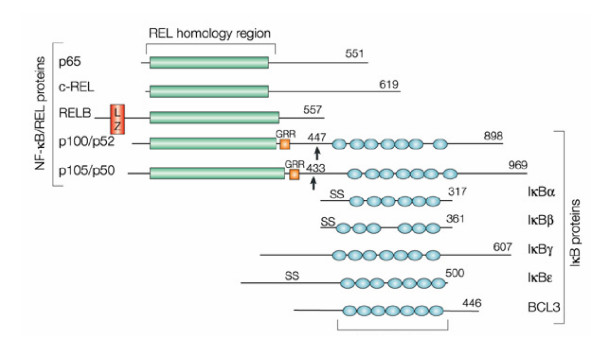
**NF-κB and IκB proteins**. A schematic representation of various domains in (Rel)/nuclear factor of kappa B (NF-κB) proteins including the Rel Homology Domain, RHD, which comprises the DNA binding domain, nuclear localization signal (NLS), dimerization domains and the IκB binding domain. (Rel)/nuclear factor of κB (NF-κB) proteins include those that do not require proteolytic processing and those that do require proteolytic processing. The first group consists of: RelA (known as p65), c-Rel and RelB and the second group includes NF-κB1 (known as p105) and NF-κB2 (known as p100), which further produce p50 and p52 proteins, respectively. These two groups dimerize, the most commonly detected NF-κB dimer is p50 – RelA. RelA is responsible for most of NF-κB transcriptional activity due to the presence of a strong transcriptional activation domain. p50 – c-Rel dimers are less abundant. Both p50 – RelA and p50 – c-Rel dimers are regulated by interactions with the inhibitor of κB (IκB) proteins, which cause their cytoplasmic localization. RelB, however, mostly associates with p100 and the p100 – RelB dimers are exclusively cytoplasmic. Proteolytic processing of p100 results in the release of p52 – RelB dimers, which translocate to the nucleus. RelB, unlike RelA and c-Rel, can function as an activator or repressor (Reproduced with permission from Nature Reviews Cancer (Vol 2, No. 4, pp 301#150;310 copyright (2002) Macmillan Magazines Ltd.).

The first identified member of the nuclear factor-kappa (κ)B (Rel/NF-κB) family was a protein found to be associated with a decameric oligonucleotide sequence in the enhancer element of the immunoglobulin kappa light chain in B-lymphocytes [[37,38], and [39]]. The Rel/NF-κB family is now known to be made up of a plethora of transcriptional regulators which share a 300 amino acid terminal domain called the Rel homology domain (RHD) [40,41]. This RHD comprises the DNA binding domain, nuclear localization signal (NLS), dimerization domains and the IκB binding domain (See Figure 3) [42,43]. Members of this family include [37]:

1. NF-κB, including p50, p65, p105 (mice devoid of *p65 *generated by targeted "knock-out" gene disruption resulted in defects in fetal development localized to the spleen and liver. However, knock-out mice devoid of *p105/p50 *expression showed no defects during their development).

2. Lyt-10 (p100), including p100 and p52, which are required in spleen development.

3. c-rel (knock-out mice showed defects in the proliferation of B and T cells).

4. relB (knock-out mice showed defects in thymus development).

5. Dorsal, which is involved in the formation of the dorsal-ventral axis of the fruit fly Drosophila [42].

Rel/nuclear factor of kappa B (NF-κB) proteins include those that do not require proteolytic processing and those that do require proteolytic processing. The first group consists of: RelA (known as p65), c-Rel and RelB. The second group includes NF-κB1 (known as p105) and NF-κB2 (known as p100), which further produce p50 and p52 proteins, respectively (See Figure 3). Members of these two groups pair with each other with the most commonly detected NF-κB being a heterodimer of p50 and RelA. RelA is responsible for most of NF-κB's transcriptional activity due to the presence of a strong transcriptional activation domain at its C-terminus. p50-c-Rel dimers are less abundant.

Both p50-RelA and p50-c-Rel dimers are regulated by interactions with the inhibitor of κB (IκB) proteins, which cause their cytoplasmic localization. RelB, however, mostly associates with p100 and the p100-RelB dimers are exclusively cytoplasmic. Proteolytic processing of p100 results in the release of p52-RelB dimers, which then translocate to the nucleus. RelB, unlike RelA and c-Rel, can function as an activator or repressor [37,43]. Of the above-mentioned proteins, only p50 and p52 are produced from the cytoplasmic precursors p105 and p100, in the presence of ATP as an energy source [43]. However, the other members contain trans-activation domains and can act as activators or inhibitors of transcription based on dimers containing or lacking trans-activation domains (See Figure 3) [44].

**Figure 4:**
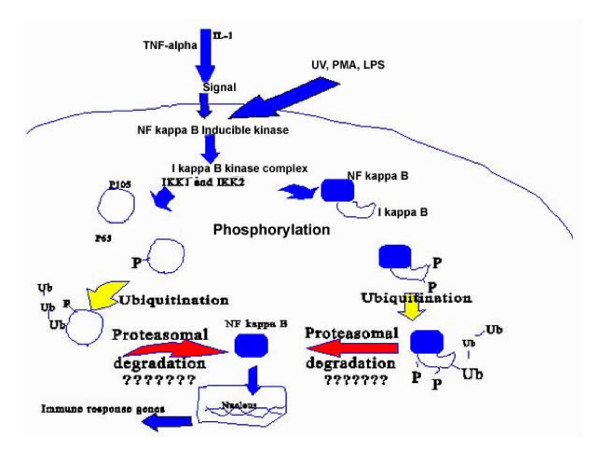
**The steps involved in the activation of NF-κB family of transcription factors (Reproduced from Ponnappan, 1998, Feb 01; 3:d152-68 with permission from Frontiers in Bioscience)**. Activators of NF-κB like TNF-alpha, PMA, UV or LPS activate the NF-κB inducible kinase, which in turn phosphorylates at least IKK1 (I kappa B kinase-alpha) and sometimes IKK2 (I kappa B kinase-beta) in the I kappa B-kinase complex. Activators of NF-κB may directly activate the kinase complex as well. This may be followed by phosphorylation of the p105/p65 complex by the kinase complex, which is in turn followed by ubiquitination, proteasomal degradation and the nuclear translocation of NF kappa B. Inside the nucleus; NF kappa B promotes the transcription of immune response genes. The "??????" indicates the possibility of lowered translocation and consequent activation of NF-κB, which occurs in various diseases.

The functions of the Rel/NF-κB family of proteins are strongly related to the target genes that contain the response elements for the protein [[37,45,46], and [47]]. For example, κB response elements are localized in *IL-2, IL-2R, Ig κ *and *MHC Classes (I) and (II) genes*, and here the Rel/NF-κB family of proteins function in modulating the immune system responses by binding to these target sequences and recruiting other immune system and inflammatory reaction mediators (See Figure 4 and Table 2). However, the Rel/NF-κB family of proteins is also directly involved in inflammatory reactions and acute phase responses when the κB binding sites are found in the regulatory sequences for the *IL-1, IL-6, TNF-α, TNF-β *and *serum amyloid A protein *genes. Also, the Rel/NF-κB family of proteins is involved in viral infections when the κB sites are found in the HIV-LTR, SV 40, CMV and adenovirus. Other functions of Rel/NF-κB proteins include growth regulation, immune system responses and cell adhesion molecules (see Table 2).

**Table 2:** Localization of κB binding motifs in the body suggests the functions of Rel/NF-κB [37].

κB sites	Related functions
IL-2, IL-2R, Igκ, MHC Classes I and II	Immune system reaction and responses.
IL-1, IL-6, TNF-α, TNF-β, serum amyloid A protein	Inflammatory reactions and acute phase responses.
HIV-LTR, SV 40, CMV, adenovirus	Viral infections
Rel/NF-κB family (NF-κB1, NF-κB2, c-rel, RelB)	Immune system responses.
p53, c-Myc, Ras, pRB1	Growth regulation.
IκB-α, IκB-γ, p105, p100 and Bcl-3	IκB family members
I-CAM, V-CAM, E-selectin, ELAM1	Cell adhesion molecules

Activation of NF-κB transcription involves the translocation of NF-κB proteins to the nucleus as illustrated in Figure 4 [[37,42,43,48] and [49]]. The factors involved in the transcriptional activation of different members of the Rel/NF-κB family are mentioned in Table 3.

**Table 3:** The factors associated with activation of NF-κB transcription factor [37] and [42].

• Cytokines (TNF-α, IL-1, IL-2, IL-6)
• Bacterial lipopolysaccharides
• Phytohemagglutinin (PHA)
• Cross-linking surface CD2, CD3, CD28 and T-cell receptors.
• Proteins secreted by viruses, for example, tax, X, E1A
• Viral infections, for example, HIV-1, Hepatitis B, HSV, HTLV-1
• Antigenic stimulants for the T and B-cells receptors
• Ultraviolet light exposure
• X-irradiation
• Nitric oxide
• Hydrogen peroxide and other oxidizing agents
• Calcium ionophores

### F. IκB inhibitor system

The multiple targets of Rel/NF-κB proteins and their multiple modes of regulation indicate that this family possesses diversity in function. Interestingly, their major mode of regulation appears to be well conserved through the IκB inhibitor system. IκB is a protein of 60–70 kDa [[38] and [43]]. The IκB inhibitor system comprises seven molecules IκB-α, IκB-β, IκB-γ, Bcl-3, p105, p100 and I κB R [45]. The inhibitor of κB (IκB) kinase (IKK) complex is composed of two catalytic subunits, IKKα and IKKβ, and one regulatory subunit, IKKγ.

**Figure 5:**
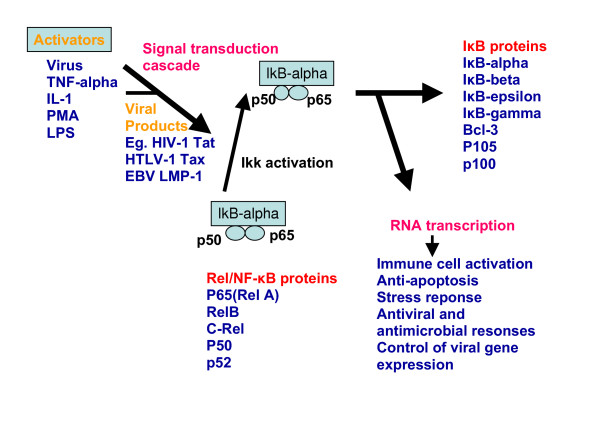
**Illustration of the Rel/NF-κB pathway**. In response to stimuli such as tumour-necrosis factor-α (TNF-α), CD40 ligand (CD40L), interleukin-1 (IL-1) or lipopolysaccharide (LPS), the IKKβ subunit is activated, and phosphorylates the IκB proteins (bound to the NF-κB heterodimers) at two conserved serines. This phosphorylation event triggers the ubiquitin-dependent degradation of IκB by the 26S proteasome, resulting in the nuclear translocation of RelA – p50 (or c-Rel – p50) heterodimers and transcriptional activation of target genes [48].

The IκB inhibitor system regulates NF-κB (p50, p65) by retaining it as a complex in the cytoplasm [54]. As a result, the NF-κB family members remain in the cytoplasm in an inactive form. In response to stimuli such as tumour-necrosis factor-α (TNF-α), CD40 ligand (CD40L), interleukin-1 (IL-1) or lipopolysaccharide (LPS), the IKKβ subunit is activated, and phosphorylates the IκB proteins (bound to the NF-κB heterodimers) at two conserved serines. This phosphorylation event triggers the ubiquitin-dependent degradation of IκB by the 26S proteasome, resulting in the nuclear translocation of RelA-p50 (or c-Rel-p50) heterodimers and transcriptional activation of target genes (See Figure 5). In response to other stimuli, such as the TNF family members lymphotoxin B (LTβ) and BAFF, IKKα is activated to induce the phosphorylation of p100 (bound to RelB) at two serine residues at its carboxyl terminus. This phosphorylation event triggers the ubiquitin-dependent degradation of the carboxy-terminal half of p100, releasing its amino-terminal half, the p52 polypeptide, which together with its heterodimer partner, RelB, translocates to the nucleus to activate transcription [[38,43], and [45]] (See Figure 5 and Table 4).

**Table 4:** Steps involved in Rel/NF-κB activation [42].

**Steps involved in Rel/NF-κB activation**
1. Exposure to a stimulus that activates NF-κB such as UV light.
2. Degradation of IκB or its inactivation by phosphorylation by means of protein kinases (McKenzie et al., 2000).
3. Dissociation of the complex between NF-κB family members and IκB inhibitor system.
4. Translocation of NF-κB proteins to the nucleus.
5. DNA binding of NF-κB proteins.
6. Transcriptional induction by NF-κB proteins.

Rel/NF-κB family members also cooperate with other transcriptional regulators such as the non-Rel/NF-κB protein Ets-1. Recent data has provided evidence that physical interaction between Ets and NF-kappaB proteins is required for the transcriptional activity of the HIV-1 and HIV-2 enhancers [47]. These interactions represent a potential target for the development of novel immunosuppressive and antiviral therapies.

### G. Role of NF-κB in apoptosis and cell survival

The dual role of NF-κB in enhancing or inhibiting apoptosis and cell death has attracted much attention in regard to its role in carcinogenesis.

#### NF-κB involvement in apoptosis

The role of Rel/NF-κB proteins in apoptosis has been well studied [[50] and [51]]. NF-κB, for instance, was found to be activated following TNF-α-induced apoptosis in several cell lines [52]. Treatment of cell lines derived from acute B-cell leukemia and human thymocytes with etoposide was found to activate NF-κB and this activation occurred prior to the initiation of apoptosis [44]. Further evidence supporting the involvement of NF-κB in apoptosis is the presence of NF-κB binding sites in the genes encoding *IL-1β converting enzyme protease*, *c-myc*, and *TNFα*, which are all involved in apoptosis and cell death [[44] and [53]]. Also, several studies showed that p65 is involved in apoptosis. This was based on an original observation whereby inhibition of apoptosis was achieved by overexpression of a dominant-negative p65 protein [54].

#### Rel/NF-κB role in cell protection and survival

The role of Rel/NF-κB proteins in cell survival is generally associated with their ability to upregulate the expression of myc [[55] and [56]]. Myc is a protein that mediates the transcriptional activation of cyclin A and cyclin D3, which are cell cycle regulators. A decrease in the myc protein concentration in the cell has been associated with apoptosis. Another pathway that leads to high myc levels is through the stimulation of CD40, a member of TNF receptor family, which results in NF-κB activation [57] and whose stimulation has been implicated in cell survival and protection [57].

The role of Rel/NF-κB as a factor in both cancerous and normal cell survival is well documented. Recent results, for instance, have shown NF-κB to be activated in the early malignant transformation of mammary cells of the breast [58]. Furthermore, NF-κB is constitutively active in pancreatic adenocarcinoma in humans [59], in T-cell leukemia cells [60], in human breast cancer [61] and in head and neck squamous cell carcinoma cell lines [62].

In non-cancer cells, NF-κB was reported to be essential for the growth and survival of sympathetic nerve cells independently of the *de novo *protein synthesis [63]. Further evidence of severe liver degeneration was associated with lack of NF-κB activation [64]. This was based on the death of murine embryonic fibroblasts that lack detectable NF-κB DNA binding activity in response to TNF-α, LPS, IL-1 and do not show IκB kinase activity required for NF-κB activation [64].

The anti-apoptotic activity of Rel/NF-κB can be regulated by other proteins. For instance, the X chromosome-linked inhibitor of apoptosis (XIAP) induces NF-κB activation by increasing the nuclear translocation of its p65 subunit [56]. In addition, CD95, which is known as Fas and possesses an apoptotic effect, was found to stimulate NF-κB degradation by caspases [66], but when an antibody against CD95 was used, caspases were inhibited and the inducibility of NF-κB was restored [66].

#### H. Xrel3

*Xrel3 *encodes an embryonic protein found to be related to the rel family of proteins. The *Xrel3 *gene is present in the genome of the amphibian, Xenopus laevis and is expressed in and is essential for the normal development of the head of Xenopus laevis embryos [67]. *Xrel3 *is also normally expressed in the otocysts and notochord of the embryonic larval stages [67]. Interestingly, Xrel3 overexpression has been implicated in the development of epidermal tumors in embryos [[14] and [67]], but little is known about how these tumors form, or whether they have similar properties to human tumors. Investigating whether the Xrel3 protein had properties that could contribute to human cancer has been explored by many researchers [[68,69] and [70]]. By applying what is known about the role of Xrel3 in embryos to human cell lines, it may be possible to uncover new knowledge about the mechanism of Rel/NF-κB activity in general.

In addition to its ability to cause embryonic tumor formation, the rationale for studying the effects of Xrel3 in human cervical cancer cells has basis in practicality. When a DNA vector encoding tagged-Xrel3 was transiently transfected into HeLa cells, Xrel3 protein constitutively localized in the nuclei, suggesting its ability to be active constantly in mammalian cells [68]. In addition, HeLa cells do not normally express Rel/NF-κB, so the transfection of Xrel3 into these cells gives the opportunity to study the activity of an interesting Rel/NF-κB protein in a negative background [[69] and [70]]. Therefore, even though Xrel3 is not a mammalian gene, its homology to the mammalian Rel/NF-κB family indicates that it may serve as a good model for gene regulation by this family enabling us to understand the mechanism of action of the Rel/NF-κB family of transcriptional regulators in cancer cells.

### I. Cancer Chemotherapy

Many chemical agents are used in the treatment of cancer. Some can be used alone in single therapy and others have to be combined or added to other regimens for an effective outcome. The five groups of single chemotherapeutic agents are: alkylating agents, antimetabolites, plant derivatives, antitumor antibiotics and the miscellaneous group which contains the platinums, procarbazine, mitotane and gallium nitrate.

#### Platinums

The platinum-containing compounds are carboplatin, cisplatin and oxaliplatin. This group of chemotherapeutic drugs is very effective in monotherapy regimens [71]. They are the most active agents in the treatment of ovarian and cervical cancers. However, they are associated with three major drawbacks [72]:

1. Severe toxicity in the form of nephrotoxicity, ototoxicity, myelosuppression and peripheral neuropathy.

2. Narrow range of tumors upon which they are effective.

3. The development of resistance after a short period of treatment.

New approaches are now designed in an attempt to expand the mechanism of action of platinums. This is done by developing a new generation of platinum-containing compounds that exhibited a broader spectrum of activity on different tumors, lower toxicity potential as well as delayed resistance to treatment [71].

#### Cisplatin

Cis DiamminedichloroplatinII (cisplatin) is one of the platinum-containing anti-cancer agents. It can be recognized from its chemical name that the cis form is the active form of the drug. The trans form was found to possess no biologic activity [72]. The mechanism of action of cisplatin is similar to the alkylating agents, but it is not identical. Cisplatin works by promoting DNA cross-linking and chelation. Recent clinical studies have shown that improved cytotoxicity of cisplatin can be attained by increasing the exposure time of the tumor to the drug [73].

### J. Rel/NF-κB and Chemoresistance

Many researches have attempted to investigate the role that NF-κB family might have in chemotherapeutic resistance. Activation of the Rel/NF-κB was found to be associated with chemotherapeutic resistance by suppressing the apoptotic potential of the chemotherapeutic drug. Recent data demonstrate that the protection from apoptosis induced in response to carbonyloxycamptothecin (CPT-11) treatment is effectively inhibited by the transient inhibition of NF-κB in a variety of human colon cancer cell lines [74]. This might be due to the cell survival effects associated with the upregulation of Rel/NF-κB family as previously mentioned. In addition, genetic manipulation aimed at inhibiting Rel/NF-κB, was found to cause sensitization of different tumor cells, like lung cancer cells, to the effect of chemotherapeutic drugs [75]. This makes the Rel/NF-κB family an attractive set of proteins to study in chemoresistant tumors.

The urge for overcomming the resistance encountered by the prolonged usage of chemotherapeutic drugs necessitates a deeper understanding of the underlying pathways that favors cell survival and inhibits apoptosis. An investigation of the upregulation of NF-κB might be a promising field of study in this regard since Rel/NF-κB activation has been associated with chemoresistance. Regarding chemotherapy, cisplatin can be used as a monotherapy without any adjuvant chemotherapeutic drugs. Cisplatin is also used in the treatment of gynecologic cancers like ovarian cancers [72]. Previous studies have shown that the apoptotic effect induced by chemotherapy in cervical cancer involves the apoptosis factor p53 and the HPV-E6 oncogenes and might be enhanced or attenuated depending on the platinum carrier ligand [76].

Recent studies by Shehata et al. investigated the effect of *Xrel3 *overexpression on the growth of HeLa cells with and without chemotherapeutic treatment [[69] and [70]]. Results showed that Rel/NF-κB might be a possible cause of chemotherapeutic resistance encountered in cervical cancer cells. This was observed at low doses of cisplatin treatment, where larger population of the malignant HeLa cells was present as compared to control cells transfected with an empty vector. However, at high concentrations of cisplatin, the upregulated Xrel3 enhanced apoptosis synergistic with cisplatin. This implies that Xrel3, a cRel homologue, possesses a dual apoptotic and antiapoptotic effect based on the degree of stress the cell might be facing.
